# Use of Continuous Regional Anesthesia Infusion as an Opioid‐Sparing Modality in Mechanically Ventilated Patients With Acute Traumatic Rib Fractures—A Retrospective Study

**DOI:** 10.1111/aas.70140

**Published:** 2025-10-27

**Authors:** Howard Meng, Ahtsham U. Niazi, Lan Zhou, Naheed Jivraj, Lavarnan Sivanathan, Stephen Choi

**Affiliations:** ^1^ Department of Anesthesiology and Pain Medicine Sunnybrook Health Sciences Centre Toronto Canada; ^2^ Department of Critical Care Medicine Sunnybrook Health Sciences Centre Toronto Canada

## Abstract

**Background:**

Continuous regional anesthesia (CRA) techniques are used for analgesia in patients with acute rib fractures. However, there is a paucity of evidence supporting the initiation of CRA in patients receiving mechanical ventilation (MV). We therefore performed this retrospective study to assess changes in opioid consumption and the rate of liberation from MV in patients with traumatic rib fractures.

**Methods:**

Patients referred to the Acute Pain Service (January 2022–July 2023) who were mechanically ventilated with acute rib fractures were included in this study. Patients received consultation either with or without CRA. Demographic and severity of injury data were collected. Mechanical ventilator requirements, pain scores, sedation use, opioids, adjunct analgesics, neurological status, and sedation status were collected for the 24 h prior to APS consultation/CRA intervention and for 48 h afterward.

**Results:**

Forty patients were included in the study, with 18 in the non‐CRA group and 22 in the CRA group. There was a statistically significant decrease in overall opioid consumption (oral morphine equivalents) for the CRA group compared to the non‐CRA group 0–48 h postintervention (0–24 h post‐CRA [median 135 mg { 33.1‐296.6}]) versus non‐CRA 368.3 (121.5–727.9) (*p* = 0.018), 24–48 h post‐CRA (31.5 mg [11.5‐131.6] vs. non‐CRA 342.8 [99.3‐645.8]) (*p* = 0.001). There was no significant difference in rates of liberation from MV between groups.

**Conclusions:**

CRA use was associated with a decrease in opioid consumption 24–48 h after CRA intervention compared to baseline. CRA did not facilitate early liberation from MV.

**Editorial Comment:**

This retrospective study provides evidence that CRA may reduce opioid requirements in mechanically ventilated patients with rib fractures. Although CRA did not facilitate earlier liberation from ventilation, the opioid‐sparing effect is clinically relevant in this population. Larger prospective studies are warranted to define optimal timing, patient selection, and integration of CRA into critical care pathways.

## 1 Introduction

Rib fractures are identified in 10% of all trauma patients, causing a potential for significant morbidity and mortality [1]. One of the main challenges is achieving adequate analgesia for these patients, as rib fracture pain can worsen respiratory mechanics, leading to respiratory complications and the need for mechanical ventilation [2]. As a regional trauma center, patients are frequently brought to our institution as polytraumas with acute rib fractures who are already mechanically ventilated due to various evolving medical conditions, including but not limited to traumatic brain injury and pulmonary contusions. These patients often receive intravenous infusions of opioid‐based analgesia. A recent systematic review and meta‐analysis has demonstrated the use of opioids for analgesia and sedation management is strongly associated with prolonged ventilator dependence [3]. Therefore, there is a need to identify and initiate opioid‐sparing interventions in an attempt to reduce barriers to liberation from mechanical ventilation in patients with acute rib fractures.

The current practice of acute rib fracture analgesia at our institution is to initiate multimodal analgesia with or without continuous regional anesthesia (CRA) as soon as possible as a standard of care, keeping within the recommended guidelines [4, 5]. CRA, in the form of thoracic epidural, erector spinae plane block (ESP), serratus anterior block (SAB), or transversus thoracis plane block, involves the continuous infusion of local anesthetics via catheters to target the spinal nerve roots, the intercostal nerves, and its subsequent nerve distributions. Given that CRA provides superior analgesia and fewer adverse effects compared to standard oral/intravenous pharmacological management, we hypothesize the initiation of CRA will decrease opioid consumption, which may provide further insight in management options to improve time to liberation from mechanical ventilation [5]. We therefore conducted a retrospective study at Canada's largest trauma center to identify pilot data of CRA intervention in patients with rib fractures in the ICU who are receiving mechanical ventilation.

## 2 Methods

Sunnybrook Research Ethics Board approved this retrospective study (project number 6114). A priori informed consent was not required from patients as this data was abstracted from the clinical records retrospectively. New APS consultations January 2022 to July 2023 requested from the ICU team were reviewed. The period selected was a sample size of convenience and therefore a power calculation was not performed. Patients either received APS consultation and no CRA (non‐CRA group) or APS consultation with CRA (CRA group). Patients must have received mechanical ventilation in the preceding 24 h before APS consultation or CRA to be included in the study.

APS consultation included recommendations for optimization of pain management using acetaminophen, nonsteroid anti‐inflammatory medications, opioids, gabapentinoids, muscle relaxants, and low‐dose ketamine infusion. CRA interventions included unilateral/bilateral ESP block, thoracic epidural, SAB, and transversus thoracic plane block (TTP). The decision to undergo CRA was at the discretion of the APS team. Our institutional standard practice for thoracic epidural is a continuous low volume infusion (2–4 cc/h 0.3% ropivacaine with 1:400,000 epinephrine). Plane‐based catheter infusions typically are run at 2 cc/h, with an intermittent bolus of 10–15 cc at 2 h intervals using ropivacaine 0.2%. Baseline demographics data (age, sex) and traumatic injuries (ISS, presence of TBI) were collected. Pain scores, ventilator settings, use of sedatives, opioids, adjunct analgesics, neurological status, and sedation status were collected for the 24 h prior to APS consultation or CRA intervention and for 48 h afterward. This data was available via our institution's electronic medical records.

Our primary objective was to analyze whether the initiation of CRA resulted in reduced opioid consumption. Secondarily, we sought to identify differences in rates of liberation from mechanical ventilation in patients with rib fractures who did and did not receive CRA. Analgesic outcomes were collected for the 24 h prior to the 48 h following consultation/intervention, while total duration of mechanical ventilation and ICU length of stay were collected.

Opioid use was recorded and converted to Oral Morphine Equivalents (OME) per equianalgesic conversion ratios according to BCguidelines.ca (3:1 from oral to IV for both morphine and hydromorphone for consistency) [6]. Univariate analysis (Mann–Whitney test for continuous data, Chi square test [Fisher's Exact if low expected cell counts] for categorical data) was carried out to identify potential differences between populations. Opioid consumption over the 72 h period was compared between groups with the Kruskal–Wallis Test. If the overall Kruskal–Wallis Test was significant, the Mann–Whitney test was utilized for pairwise comparisons between groups for each of the 24 h periods described above. The Wilcoxon Rank test was used for within‐group comparisons. Due to the limited sample size, subgroup analysis was not carried out for the different intervention groups. Pain scores were recorded in the ICU as either Critical Care Pain Observation Tool (CPOT) or numeric rating scale (NRS). If NRS was available, the highest NRS recorded during the period was reported. CPOT 0,1,2 indicating no pain was converted to NRS 1; CPOT 3,4 indicating moderate pain was converted to NRS 5; CPOT 5,6,7,8 indicating severe pain was converted to NRS 8 based on work by Severgnini et al. and Briggs and Closs [7, 8]. NRS pain scores were analyzed between groups for each 24 h period with the Mann–Whitney test. Statistical analysis was performed with SPSS 21 (UBM, Armonk, NY).

## 3 Results

Between January 2022 and July 2023, a total of 40 patients with acute rib fractures who were mechanically ventilated in the ICU were referred to the APS for consultation. There were a total of 198 patients who had ≥ 2 rib fractures and were mechanically ventilated at our institution during the study period. Eighteen patients received APS consultation and standard management without CRA, and 22 patients received standard care with CRA. Patient demographic information and injury characteristics are reported in Table 1. Patients who received CRA were older (median 63.3 [49–79.3] vs. 48.2 [27.5–62.8], *p* = 0.03). The most common type of CRA was ESP (16 patients—11 unilateral catheters and five bilateral catheters). Patients receiving CRA had more extensive chest wall injury (≥ 5 rib fractures: 20/22 CRA group vs. 11/18 no CRA group, *p* = 0.025). There was no significant difference in ISS, presence of flail segments, pneumo/hemothorax, presence of sternal fracture, or presence of TBI.

**TABLE 1:** Patient demographic information and injury characteristics.

	CRA	No CRA	*p*
Total *n*	22	18	
Age, median [IQR]	64 [49–79.3]	49.5 [27.5–62.8]	0.03
Sex (male), *n* (%)	14 (64)	15 (83)	0.16
Traumatic injuries, *n* (%)			
≥ 5 rib fractures	20 (91)	11 (61)	0.025
Flail segment	3 (14)	5 (28)	0.27
Pnuemothorax or hemothorax	18 (82)	11 (61)	0.14
Sternal fracture	7 (31)	3 (17)	0.27
Traumatic brain injury	9 (7)	6 (33)	0.62
ISS score, median [IQR]	22 [19–30.3]	26.5 [22–35]	0.14
Rib fixation, *n* (%)	1 (5)	1 (6)	0.88
Consult and intervention (hours), median [IQR]			
Time from hospital admission to APS consultation	34.5 [15.4–53.4]	48.5 [27.4–122.1]	0.16
Time from APS consultation to CRA	18.5 [0.5–38.4]	N/A	
CRA duration (days), Median [IQR]	6 [4.8–7]	N/A	
CRA type, *n* (%)			
Erector spinae plane	11 (50)	N/A	
Bilateral erector spinae plane block	5 (23)	N/A	
Thoracic epidural anesthesia	3 (14)	N/A	
Serratus anterior plane	2 (9)	N/A	
Transversus thoracic plane	1 (5)	N/A	

Abbreviations: APS, acute pain services; CRA, continuous regional anesthesia; ISS, Injury Severity Score.

There were statistically significant differences in opioid consumption between the CRA and non‐CRA groups over 72 h (*p* = 0.001). Baseline opioid consumption in the 24 h before APS consultation was not significantly different between both groups (Table 2). Median OME consumption in the 24 h period after CRA differed between groups (135 mg [33.1–296.6] vs. 368.3 mg [121.5–727.9], *p* = 0.018). A statistically significant difference in OME was also present in the 24–48 h period (CRA 31.5 mg [11.5–131.6] vs. 342.8 mg [99.3–645.8], *p* = 0.001). There were statistically significant decreases in opioid consumption for the CRA group when comparing 24 h pre‐CRA versus 24 h post‐CRA (*p* = 0.007) and 24 h pre‐CRA versus 24–48 h post‐CRA *(p* = 0.002). No statistically significant changes were seen in opioid consumption for the non‐CRA group at any timepoint.

**TABLE 2:** Opioid consumption and pain verbal rating score (VRS) at rest and with movement before and after CRA intervention or APS Consultation.

	CRA	No CRA	*p* ^a^
Total *n*	22	18	
Oral morphine equivalent (mg), median [IQR]			
24 h pre‐CRA/consult	268.5 [121.5–386.3]	365.3 [100.5–541.3]	0.50
0–24 h post‐CRA/consult	135 [33.1–296.6]^b^	368.3 [121.5–727.9]^c^	0.018
24–48 h post‐CRA/consult	31.5 [11.5–131.6]^d^	342.8 [99.3–645.8]^c^	0.001
Worst VRS Score (at rest), median [IQR]			
24 h pre‐CRA/consult	1 [1–5]	3 [1–8]	0.35
0–24 h post‐CRA/consult	3 [1–8]	5 [1–5]	0.97
24–48 h post‐CRA/consult	1 [0–5]	1 [0–5]	0.62
Worst VRS Score (with movement), median [IQR]			
24 h pre‐CRA/consult	5 [3–8]	6 [1–8]	0.79
0–24 h post‐CRA/consult	5 [1.8–6.3]	8 [5–8]	0.03
24–48 h post‐CRA/consult	1.5 [1–5.8]	5 [5–8]	0.08

^a^ *p* value represents an unpaired *t* test for VRS Scores and a Mann–Whitney U test for OME between group comparisons.

^b^ *p* = 0.007, representing pairwise comparison Opioid Consumption at 0–24 h postintervention relative to preintervention.

^c^ No statistically significant difference was noted in pairwise comparisons between OME at measured timepoint, relative to preintervention.

^d^ *p* = 0.002, representing pairwise comparison Opioid Consumption at 24–48 h postintervention relative to preintervention.

There was no statistically significant difference in worst pain score at rest and with movement at across the 72 h time period within CRA and non‐CRA groups (Table 2). There were no significant differences in median ventilator days (6 [IQR 3–8.3] vs. 4.5 [IQR 2–11.3], *p* = 0.64) or median ICU days (10 [IQR 7.5–16.8] vs. 8.5 [IQR 6–16], *p* = 0.40) (Table 3). There was no difference in liberation from ventilator between the two groups at 0–24 and 24–48 h.

**TABLE 3:** Secondary outcomes measured among those who received CRA intervention or APS Consultation.

	CRA	No CRA	*p*
Total *n*	22	18	
Ventilation days, median [IQR]	6 [3–8.3]	4.5 [2–11.3]	0.64
Intensive care unit days, median [IQR]	10 [7.5–16.8]	8.5 [6–16]	0.40
Mechanically ventilated, *n* (%)			
24 h pre‐CRA/consult	22 (100)	18 (100)	1.00
0–24 h post‐CRA/consult	18 (82)	17 (94)	0.36
24–48 h post‐CRA/consult	12 (55)	12 (67)	0.53
Best Glasgow Coma Score, median [IQR]			
24 h pre‐CRA/consult	10 [8.8–11]	11 [10–11]	0.08
0–24 h post‐CRA/consult	11 [10–14]	11 [10–11]	0.64
24–48 h post‐CRA/consult	11 [10–14]	11 [10–14]	0.90

## 4 Discussion

To our knowledge, this is the first evidence that demonstrates the use of CRA provides a reduction in opioid consumption in a polytrauma population while mechanically ventilated. Our study demonstrated a significant reduction in opioid consumption at 24 h intervals before and after CRA without significant changes in pain scores. Our findings of opioid reduction are similar to other studies that have utilized CRA for multiple rib fractures [9]. Single injection regional anesthesia techniques, including the use of SAB to a rib fracture bundle, have also demonstrated superior pain control and significantly reduced opioid consumption [10, 11]. Our study is different in that, while there was a reduction in opioid consumption, our patients did not report less pain with CRA than those patients who had no CRA. This may be because our population sustained multiple other distracting traumatic injuries rather than isolated chest wall injury.

There have been several reviews published regarding the use of regional anesthesia for rib fractures; however, none have provided meaningful guidance on the use of CRA in patients who are mechanically ventilated [12, 13, 14]. The most relevant study to date in our population of interest has been Ramin et al.'s study comparing sedation versus CRA and sedation in patients who were mechanically ventilated with rib fractures [15]. In their group of patients with comparable ISS, there was no observed difference in sufentanil consumption in the initial 2 days on admission to hospital after CRA initiation. Notably, our study period was approximately Day 3–6 after admission, which could correspond to the completion of surgical interventions for other traumatic injuries and therefore a reduction in other distracting pains and opioid consumption. The use of CRA during our study period might better reflect the true effect of CRA on rib fracture in the mechanically ventilated.

Despite the reduced opioid consumption following CRA, our study did not demonstrate a difference in earlier liberation from mechanical ventilation in the CRA group. Successful liberation from mechanical ventilation requires a coordinated interdisciplinary approach that includes targeted analgesia using CRA, sedation holidays, spontaneous breathing trials, and treatment of other factors impacting ventilation, that is, antibiotic therapy for pneumonia. Initiation of CRA and subsequent reduction in opioid consumption without changes in other practice may reflect the lack of difference in earlier rate of liberation from mechanical ventilation. Challenges in tracheal extubation may be secondary to severity of chest injury in which underlying pulmonary contusion may result in poor lung function [16]. Traumatic brain injury may also present challenges with liberation from mechanical ventilation although there was not a significant difference in the number of TBI patients in each of our cohort [17]. In fact, the patients in the CRA group had significantly worse neurological status based best GCS in the 24 h prior to intervention.

As a regional trauma center, we often manage patients with acute rib fractures receiving mechanical ventilation. These patients were sometimes delayed in transfer to our institution and therefore did not receive CRA on arrival. Opioid‐based sedation is the standard of practice at our institution for these patients and poses a major concern due to its association with prolonged mechanical ventilation. The timing of CRA in this population is unclear, with one side arguing that CRA should not be initiated until weaning from mechanical ventilation begins, whereas others would argue that opioid‐sparing techniques have the potential to reduce opioid‐related adverse effects and sequelae [18]. Other risks concerning opioid use in the critically ill include delirium, ICU‐acquired infections due to immunosuppression, tolerance, and hyperalgesia. In the long term, suggested risks include persistent opioid use, chronic pain following ICU discharge, and posttraumatic stress disorder [19]. Therefore, reducing opioid consumption during mechanical ventilation is crucial in minimizing complications.

There are several limitations to this study. (1) The retrospective nature of the study restricts our ability for more extensive data collection. (2) There are limited patient numbers included in this study and therefore are likely underpowered to detect statistically significant differences in the rate of liberation from mechanical ventilation and in pain scores. (3) There was selection bias in patients requested for consultation from APS as there was no established protocol and it represented approximately 1/5th of the total available patients during the study period. (4) The trauma population is a heterogenous population with potential for confounding injuries which can mask the true effect of CRA in this population. (5) There was a lack of coordination between APS and ICU teams in patients with CRA for sedation holiday and spontaneous breathing trials. (6) There was patient selection bias to initiate CRA based on timing (less likely if rib fractures occurred > 1 week) and severity of rib fracture pain in relation to other concomitant injuries. (7) The study is nonrandomized, so there is potential imbalance in known and unknown prognostic factors. (8) Additional data such as additional surgeries, sedation level/dose, and delirium duration were not included and therefore limit our interpretation of our results. (9) Data was not collected on complications of CRA. Our intent was not to provide definitive evidence on the use of CRA in mechanically ventilated rib fracture patients but to generate preliminary data to indicate that benefit may exist and guide future study design given our institution's experience as the largest trauma center in Canada.

## 5 Conclusions

In this group of mechanically ventilated patients with acute rib fractures, the initiation of CRA resulted in a significant decrease in opioid consumption at 24 and 48 h, which has not been previously established. Given that the significant findings of opioid reduction were approximately 3–6 days after admission, future studies should be designed over a longer period to be representative of the entirety of the acute care period. The clinical relevance of opioid reduction in this context requires further investigation in the form of a larger, prospective, randomized study.

## Author Contributions

H.M., A.U.N., L.Z., and S.C. provided substantial contributions to the conception or design of the study. H.M., A.U.N., and L.Z. acquired the data for the study. N.J., L.S., and S.C.analyzed and interpreted the data. All authors participated in drafting the manuscript; H.M. revised it critically. All authors read and approved the final version of the manuscript. All authors contributed equally to the manuscript and read and approved the final version of the manuscript.

## Conflicts of Interest

S. Choi receives nonfinancial support from CogState LLC (no cost access to cognitive testing software). The other authors declare no conflicts of interest.

## Data Availability

The data that support the findings of this study are available from the corresponding author upon reasonable request.
